# UTP Regulates the Cardioprotective Action of Transplanted Stem Cells Derived From Mouse Cardiac Adipose Tissue

**DOI:** 10.3389/fphar.2022.906173

**Published:** 2022-06-15

**Authors:** Esteban Diaz Villamil, Lucas De Roeck, Marion Vanorlé, Didier Communi

**Affiliations:** Institute of Interdisciplinary Research, IRIBHM, Université Libre de Bruxelles, Brussels, Belgium

**Keywords:** P2Y receptors, adipose-derived adult stem cells, cardiac ischemia, extracellular nucleotides, cardioprotection

## Abstract

Adipose tissue is a source of stem cells with a high potential of differentiation for cell-based regenerative therapies. We previously identified mouse P2Y_2,_ an ATP and UTP nucleotide receptor, as a regulator of adipogenic and endothelial differentiation of cardiac adipose-derived stem cells (cADSC). We investigated here the potential involvement of P2Y_2_ receptor in the cardioprotective action of undifferentiated cADSC transplantation in mouse ischemic heart. Transplantation of cADSC was realized in the periphery of the infarcted zone of ischemic heart, 3 days after left anterior descending artery ligation. A strong reduction of collagen stained area was observed 14 days after cADSC injection, compared to PBS injection. Interestingly, loss of P2Y_2_ expression totally inhibits the ability of transplanted cADSC to reduce cardiac fibrosis. A detailed gene ontology enrichment analysis was realized by comparing RNA-sequencing data obtained for UTP-treated wild type cASDC and UTP-treated P2Y_2_-null cASDC. We identified UTP target genes linked to extracellular matrix organization such as matrix metalloproteinases and various collagen types, UTP target genes related to macrophage chemotaxis and differentiation into pro-fibrotic foam cells, and a significant number of UTP target genes linked to angiogenesis regulation. More particularly, we showed that UTP regulated the secretion of CCL5, CXCL5, and CCL12 chemokines and serum amyloid apolipoprotein 3, in the supernatants of UTP-treated cADSC. Interestingly, CCL5 is reported as a key factor in post-infarction heart failure and in the reparative and angiogenic action of transplanted ADSC on ischemic tissue. We investigated then if a UTP-pretreatment of cADSC amplifies their effect on cardiac revascularization in mouse ischemic heart. Transplantation of cADSC was able to increase peri-infarct capillary density, 14 days after their injection. This beneficial effect on cardiac revascularization was enhanced by a UTP-pretreatment of cADSC before their transplantation, and not observed using P2Y_2_-null cADSC. Our data support that the efficacy of transplanted cADSC can be regulated by the release of inflammatory mediators such as extracellular nucleotides in the ischemic site. The present study highlights the P2Y_2_ receptor as a regulator of cADSC cardioprotective action, and as a potential target for the therapeutic use of undifferentiated cADSC in post-ischemic cardiac ischemia.

## Introduction

Adipose-derived stem cells (ADSC) are now recognized as an ideal source for therapeutic applications due to their multidifferentiation capacity and low immunogenicity (Schenke-Layland et al., 2009). ADSC implanted in the myocardium after ischemic injury produce and release pro-angiogenic, anti-apoptotic and anti-inflammatory cytokines and growth factors (Lévy et al., 2013; Zhao et al., 2017).

The therapeutic effects of ADSC are caused more by their secretory potential than by their cardiac differentiation capacity and direct integration into damaged tissue (Gnecchi et al., 2008). It is thus essential to elucidate the paracrine mechanisms underlying tissue repair and regeneration after ADSC transplantation. Pretreatment of ADSC before transplantation with specific factors constitutes a major approach to improve ADSC cardioprotective therapeutic effects (Stubbs et al., 2012; Zhu et al., 2015). Nevertheless the optimization of the cardioprotective abilities of transplanted ADSC in myocardial infarction treatment remains a major issue. ADSC isolated from cardiac adipose tissue display a better potential to differentiate into multiple cardiovascular cells including cardiomyocytes, endothelial cells, and vascular smooth muscle cells *in vitro* than stem cells derived from other fat depots such as subcutaneous and visceral adipose tissues (Nagata et al., 2016).

Therapeutic treatments for myocardial infarction display a limited success in preventing the progression of left ventricular remodeling, which results in only a partial restoration of cardiac function (Zhu et al., 2015; Zhao et al., 2017). Many research studies and clinical trials have highlighted the ability of ADSC injection to reduce myocardial infarction, promote post-ischemic vascularization and improve cardiac function (Yang et al., 2013; Chen et al., 2014). The use of ADSC has reinforced the idea that stem cell transplantation is still a promising strategy of therapeutic revascularization developed for patients with myocardial infarction (MI) (Cai et al., 2009). The regulation of cardiac fibrosis as well as revascularization and restoration of blood flow are the basis of the used therapeutic interventions to myocardial ischemia. Therapy based on the use of adipose-derived stem cells has already been performed for tissue regeneration and revascularization. Their paracrine actions include pro-angiogenic and anti-apoptotic effects through the secretion of various cytokines (Nagata et al., 2016) and their efficacy has been already tested in clinical trials (Cai et al., 2009; Chen et al., 2014).

An important release of extracellular nucleotides by cardiomyocytes and cardiac endothelial cells has been reported during myocardial ischemia (Dutta et al., 2004) and could modulate the inflammatory and fibrotic response within the infarcted area. P2Y_2_ receptor is an ubiquitously expressed ATP/UTP G-protein-coupled nucleotide receptor coupled to the phosphoinositide pathway (Lustig et al., 1993). P2Y_2_ activation significantly reduces cardiomyocyte death induced by hypoxia and UTP administration to rats reduces infarct size (Yitzhaki et al., 2005). We demonstrated previously that loss of P2Y_4_ receptor, another ATP/UTP nucleotide receptor, is associated with a protection against infarction and a decrease in cardiac inflammation, fibrosis and permeability (Horckmans et al., 2015). Purinergic receptors are considered as key regulators of proliferation, differentiation, cell death, and engraftment of diverse stem cells (Glaser et al., 2012; Zippel et al., 2012; Kaebisch et al., 2015). We demonstrated the differential involvement of P2Y_2_ and P2Y_4_ receptors on adipogenic differentiation of mouse cardiac adipose-derived stem cells (cADSC) (Lemaire et al., 2017; Negri et al., 2019). More recently, we showed that UTP is a regulator of endothelial differentiation and angiogenic properties of cADSC (Vanorlé et al., 2021).

Therapeutic applications based on ADSC preconditioning with extracellular nucleotides may provide a new advance for cardiac tissue repair. In the present study, we investigated the potential role of P2Y_2_ receptor in the cardioprotective action of transplanted undifferentiated cADSC in an *in vivo* model of mouse myocardial infarction.

## Methods

### Animals

P2Y_2_ knockout (KO) CD1/C57BL/6J mice were a kind gift from Dr. B. Koller (University of North Carolina, Chapel Hill, United States) (Homolya et al., 1999). 12- to 16-week-old male and female wild type (WT) and P2Y_2_ KO mice were used randomly for cADSC isolation and ischemia experiments.

### cADSC Isolation and Culture

cADSC were isolated from the stromal vascular fraction of wild type (WT) and P2Y_2_ KO mouse cardiac adipose tissue and their purity was checked by flow cytometry as previously described (Nagata et al., 2016). The cardiac adipose tissue, located between the visceral pericardium and the epicardium (including epi- and pericardic fat depots) was freshly harvested. Adipose tissue was washed in phosphate-buffered saline (PBS) and minced, followed by a digestion in collagenase A (2.5 g/L) at 37°C for 45 min. The digested tissue was filtered through a 100 μm cell strainer (Corning) and centrifuged at 500 g for 5 min. The supernatant containing adipocytes and debris was discarded. A red blood cell lysis was performed on the pelleted cells by an osmotic shock using ammonium-chloride-potassium (ACK) lysing buffer containing 0.15 M ammonium chloride, 0.01 M potassium bicarbonate and 0.0001 M disodium EDTA. After a last centrifugation at 500 g for 5 min, the resultant pellet, named the stromal vascular fraction, was suspended in Dulbecco’s modified Eagle’s medium (DMEM, Gibco) supplemented with 10% fetal bovine serum and 1% penicillin-streptomycin, plated at a density of 5 × 10^4^ per cm^2^ and incubated at 37°C in a humidified 95% O_2_/5% CO_2_ atmosphere. cADSC were selected by their high plastic adhesion, non-adherent cells were removed after 24 h, and the culture medium was subsequently replaced every 2 days. The cADSC cultures were not stimulated or stimulated daily with UTP (100 µM) for 7 days in the DMEM medium described above. A fraction of cADSC was used to check their purity before transplantation by flow cytometry using CD90, C105, and CD44 markers, as previously described (Nagata et al., 2016).

### Myocardial Infarction Model and Intra-Myocardial cADSC Injection

WT mice were anesthetized with a mixture of Dormazolam (midazolam, 5 mg/kg), Domitor (medetomidine hydrochloride, 0.5 mg/kg) and Fentadon (fentanyl, 0.05 mg/kg), intubated and mechanically ventilated (rate 130 stroke/min, tidal volume 0.13 ml). Optical magnification loop was used for better visualization of the operation field. A left side thoracotomy was performed between the third and the forth rib, and the pericardium was incised. Once the heart was exposed, MI was induced by the permanent ligation of the left anterior descending artery (LAD) proximal to its bifurcation from the main stem. Successful performance of coronary occlusion was confirmed by blanching of the myocardium distal to the coronary ligation. The thoracic incision was then closed with a 5-0 silk suture at the muscle tissue and a 7-0 silk suture at the skin. An antagonist cocktail of Naloxon (naloxone hydrochloride, 1.2 mg/kg), Anexate (flumazenil, 0.5 mg/kg) and Atipam (atipamezole hydrochloride, 2.5 mg/kg) was finally administered to the mice to reverse the general anesthesia. An intraperitoneal injection of Temgesic (buprenorphine, 0.3 mg/kg) was administered after the surgery and the next morning. Three days after LAD, mice underwent a second thoracotomy, as described above. This was followed by intramyocardial cADSC injections. Briefly, cADSC cultured in DMEM for 7 days, untreated or stimulated daily with UTP (100 µM), were detached with trypsin/EDTA and resuspended in PBS. These cells were then injected at a concentration of 10^4^ cells/µl at 3 different sites along the infarct border zone with a final volume of 10 μl at each site (3 × 10^5^ cells/heart). Sham/control group mice underwent the same experimental procedure excepted that they were subjected only to PBS injection. 14 days after injection, hearts were perfused with PAF 4%, collected, fixed in PAF 4%, and either immerged in isopropanol 70% and embedded in paraffin or immerged in a 10%, 20%, and finally a 30% sucrose solution and embedded in Tissue-Tek OCT compound (Sakura Finetek) and frozen at −80°C.

### Quantification of Fibrosis Area in Ischemic Hearts

Paraffin cross-sections (8 µm) of infarcted hearts were cut, fixed in Bouin’s solution (Sigma-Aldrich) and stained with Masson’s trichrome (Sigma-Aldrich), according to the manufacturer’s protocol. Sections were counterstained with hematoxiline and mounted. Images of whole hearts were acquired with NanoZoomer-SQ (Hamamatsu) at 0.23 μm/pixel. Fibrosis was quantified as the relative area of the blue staining (collagen) on five sections per ischemic heart compared to the left ventricle surface, using ImageJ software.

### Quantification of Vascular Density in Ischemic Hearts

The vascularity of the ischemic myocardium was assessed by staining with specific markers recognizing the presence of capillaries and arterioles. Briefly, 8 µm-thick heart cryosections were stained with an anti-isolectin B4 antibody—Alexa Fluor ™ 647 conjugate (1/400 ON at 4°C) (Invitrogen, Merelbeke, Belgium), an anti-α-smooth muscle actin antibody—Cy3™ conjugate (1/400 1 h at RT) (Sigma-Aldrich, St. Louis, MO, United States) and Hoechst (1/2000 1 min at RT) (Thermo Scientific, Merelbeke, Belgium). The area of the infarct border zone was determined as the 0.5 mm region of histologically intact myocardium surrounding the infarct-related fibrocellular region. Capillary and arteriole density was quantified in this peri-infarct myocardium. The final data were expressed as the number of capillaries or arterioles (<20 µm in diameter) per square millimeter, the percentage is referring to the ratio between control condition (PBS injection) and cADSC injection conditions. These analyses were performed using ImageJ software by examining 30 counting surfaces/field for capillary and arteriole density on a total of five sections per heart, at ×10 magnification, in a blinded fashion. Sections were counterstained with Hoechst to visualize the entire population of cell nuclei within each myocardial section.

### Image Acquisition of Transplanted Ischemic Hearts

Images were acquired at LiMiF (Université Libre de Bruxelles, Faculté de Médecine, Bruxelles, Belgique), on an Axio Observer Z1 inverted microscope (Zeiss) equipped with a Zeiss Axiocam 702 mono camera using a EC Plan NeoFluar ×10/0.3 dry objective (Zeiss). The microscope is equipped with an HBO 100 light source. Three channels were recorded with narrow band-pass filter sets (Zeiss) #49 (ex. 335–383 nm, em. 420–470 nm), #43 (ex. 538–562 nm, em. 570–640 nm), and #50 (ex. 625–655 nm, em. 665–715 nm) used to visualize blue, red and far-red fluorochromes, respectively. Images of 2.3 pixels (Axiocam 702 m) were acquired and recorded as 16-bit czi files. The field of view is defined by the ×10 objective resulting in 1.13 mm by 712.58 micron images. The pixel scaling results in 0.586 micron by 0.586 micron. Settings were kept identical for all conditions. Single plane images were displayed using Zen (Blue Edition) software (Zeiss) and exported as uncompressed TIF images.

### RNA-Sequencing Experiments

RNA-sequencing experiments were performed on WT or P2Y_2_ KO cADSCs cultured in DMEM for 7 days, untreated or treated every 24 h with UTP (100 µM). RNA was extracted using the RNeasy Mini Kit (Qiagen) after cell lysis using TRI Reagent Solution (Invitrogen). 1 µg/50 µl of RNA was engaged and the quality was checked using a Bioanalyzer 2100 (Agilent technologies). RNA-sequencing experiments were performed on two different pools of RNA from two cADSC cultures per condition. Complementary DNA (cDNA) libraries were obtained using the TruSeq Stranded mRNA Library Prep kit (NuGen) following manufacturer recommendations. The multiplex libraries (18 pM) were loaded on flow cells and sequences were produced using a HiSeq PE Cluster Kit v4 and TruSeq SBS Kit v3-HS from a HiSeq 1500 (Ilumina). Approximately 25 million paired-end reads per sample were mapped against the mouse reference genome (GRCm38.p4/mm10) using STAR software to generate read alignments for each sample. Annotations Mus_musculus GRC38.87.gtf were obtained from ftp.Ensembl.org. After transcripts assembling, gene level counts were obtained using HTSeq.

A gene enrichment analysis was performed with DAVID software on the RNA-sequencing data to determine which biological processes were enriched for differentially expressed genes. Only genes with CPM > 0.5 and a fold change >2 or <0.5 were considered. The modified Fisher Exact *p*-value or EASE score was reported by the software and indicated as logarithmic *p*-value.

### Quantitative Reverse-Transcription Polymerase Chain Reaction

Total mRNA was extracted from cADSC, cultured for 7 days in DMEM and untreated or treated daily with UTP (100 µM), as described above. RNA samples were eluted with RNase-Free water and RNA concentration was quantified by NanoDrop. Reverse transcription was performed with 500 ng mRNA, 0.5 µl random hexamers, 1 µl dNTPs (10 mM), 4 µl ×5 First Strand Buffer (250 mM Tris-HCl, pH 8.3, 375 mM KCl, 15 mM MgCl_2_), 2 µl DTT (0.1 M), 1 µl RNaseOUT ribonuclease inhibitor (40 U/µl), 1 µl SuperScript II reverse transcriptase (200 U/µl) and RNase-Free water up to a volume of 20 µl. Samples were incubated at 42°C for 50 min to synthesize cDNA and at 70°C for 10 min to stop the reaction. Quantitative polymerase chain reaction (qPCR) experiments were performed using 5 ng cDNA, specific primers (5 µM) for UTP target genes and P2Y receptors, 2x KAPA SYBR FAST qPCR Master Mix [KAPA SYBR FAST DNA Polymerase, reaction buffer, dNTPs, SYBR Green I dye, and MgCl2 (2.5 mM)], 50x KAPA SYBR FAST ROX Low and RNase-Free water up to a 20 µl volume on a 7500 Fast Real-Time PCR System (Applied Biosystems). Expression values for each gene were normalized to the expression of the housekeeping gene Rpl32. Results were expressed as 2^−ΔCt^. The specific primers for Ccl5, Cxcl5, Ccl12, Saa3, and Rpl32 genes and for P2ry2 and P2ry4 genes are shown in Table 1.

**TABLE 1:** Specific primers for UTP target genes, P2Y receptor genes and Rpl32 gene. Specific primers for UTP target genes Ccl5, Cxcl5, Ccl12, and Saa3, and for P2ry2 and P2ry4 receptor genes, were used in qPCR experiments and normalized to Rpl32 mRNA level.

Gene	Forward primer sequence	Reverse primer sequence
Ccl5	5'-GCT​GCT​TTG​CCT​ACC​TCT​CC-3′	5′-TCG​AGT​GAC​AAA​CAC​GAC​TGC-3′
Cxcl5	5′-CCT​CCT​TCT​GGT​TTT​TCA​GTT​TAG​C-3′	5′-GCA​TTC​TGT​TGC​TGT​TCA​CGC​TG-3′
Ccl12	5′-AGT​CCT​CAG​GTA​TTG​GCT​GG-3′	5′-CTT​CCG​GAC​GTG​AAT​CTT​CT-3′
Saa3	5′-GTT​GAC​AGC​CAA​AGA​TGG​GT-3′	5′-CCC​GAG​CAT​GGA​AGT​ATT​TG-3′
P2ry2	5′-AGC​CCT​TGT​ACT​GCG​CAA​AAC-3′	5′-GAA​GAT​ATA​GAG​AGC​CAC​GAC​GTT-3′
P2ry4	5′-GCC​CAA​GTT​CTG​GAG​ATG​GTG-3′	5′-GGT​GGT​TCC​ATT​GGC​ATT​GG-3′
Rpl32	5′-GCA​CCA​GTC​AGA​CCG​ATA​T-3′	5′-CAG​GAT​CTG​GCC​CTT​GAA​C-3′

### ELISA Experiments

Supernatants from cADSC cultured in DMEM for 7 days, untreated or treated daily with UTP (100 µM), were collected. CCL5, CXCL5, CCL12, and SAA3 protein levels were quantified by ELISA following the manufacturer’s procedure (R&D Systems). Total proteins were quantified using Pierce™ 660 nm Protein Assay Reagent (Thermo Scientific) supplemented with Ionic Detergent Compatibility Reagent (Thermo Scientific) to normalize ELISA results.

### Statistical Analysis

All data are expressed as mean ± SEM, and statistical analysis was performed with Prism Software (version 6; GraphPad, San Diego, CA, United States). Endpoint comparisons between 2 groups were performed using unpaired 2-tailed Student’s t-test. For multiple comparisons, false discovery rate according Benjamini-Hochberg was applied to control for type I false positive errors, FDR was set to 0.05. For parallel repeated-measures studies, 2-way ANOVA was used with Bonferroni post-hoc evaluation to determine the significance for individual time points. A 2-tailed *p* < 0.05 was considered as significant.

## Results

### Role of P2Y_2_ Receptor in the Ability of Transplanted cADSC to Reduce Post-ischemic Cardiac Fibrosis

Stromal cells were isolated from cardiac adipose tissue of WT and P2Y_2_ KO mice. Non-adherent cells were removed 24 h after the plastic adherence step. Flow cytometry experiments were performed with adherent stromal cells using CD90, CD105, and CD44 as markers of mesenchymal stem cells, and CD26 as fibroblast marker. More than 90% of isolated cells were CD90^+^ CD105^+^ CD44^+^ CD26^−^, and they were cultured for 7 days in proliferation medium (data not shown). These cADSC were then counted for intramyocardial injections (3 × 10 µl containing 10^5^ cells each). LAD ligation was performed on WT mice and intramyocardial injections of PBS (control), WT cADSC and P2Y_2_ KO cADSC were performed in the peri-infarct border zone, 3 days after LAD ligation. 14 days after injection, hearts were harvested and embedded in paraffin for further analysis.

**FIGURE 1:**
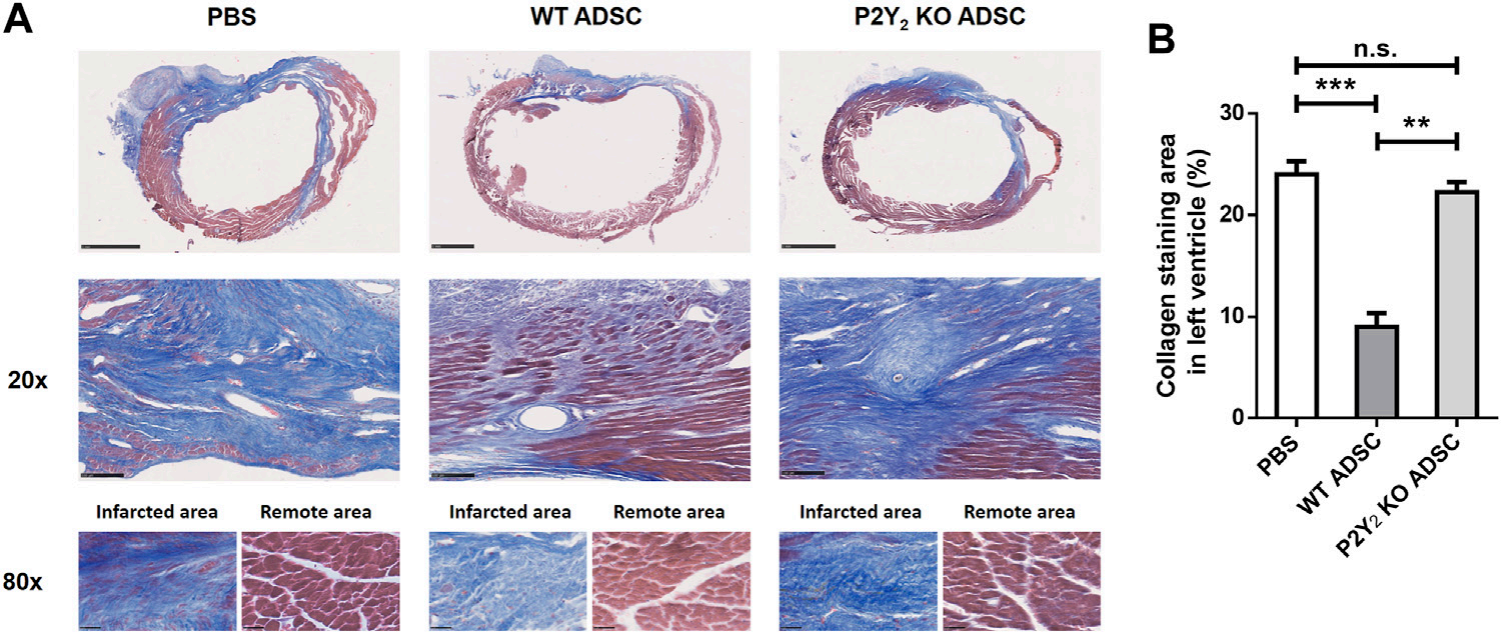
Role of P2Y_2_ receptor in the transplanted cADSC ability to reduce post-ischemic cardiac fibrosis. **(A)** Representative Masson’s trichrome staining of cardiac fibrosis in transplanted ischemic hearts. PBS, WT cADSC or P2Y_2_ KO cADSC were injected in the peri-infarct zone of ischemic hearts, 3 days after LAD ligation. 14 days after injection, images of Masson’s trichrome staining (in blue) in ischemic hearts were obtained for total heart (scale bar represents 1 mm), at ×20 magnification for the fibrotic area (scale bar represents 100 µm) and at ×80 magnification inside the infarcted and remote area regions (scale bar represents 25 µm) **(B)**. Quantification of fibrosis area normalized to total left ventricle area in ischemic hearts, 14 days after injection of PBS, WT cADSC or P2Y_2_ KO cADSC. Fibrosis area was quantified as the relative surface of collagen blue staining on five sections per ischemic heart, quantified by color image analyzer ImageJ in left ventricle (LV) and expressed as percentages of total LV surface (*n* = 3–5). Data represent mean ± SEM. *p < 0.05; **p < 0.01; ***p < 0.001, n.s., not significant.

Five heart sections per transplanted ischemic heart were stained with Masson’s trichrome to evaluate cardiac fibrosis. Fibrosis was quantified by calculating the area stained blue, expressed as a percentage of the left ventricle’s total area (Figures 1A,B). We observed a strong reduction of cardiac fibrosis in ischemic heart transplanted with WT cADSC compared to mice injected with PBS. Collagen stained area was reduced from 24.0 ± 1.3% of left ventricle area in PBS-injected hearts to 9.0± 1.3% of left ventricle area in WT cADSC-injected hearts (decrease of 62.5 ± 8.5%; mean ± SEM; ***: *p* < 0.001) (Figure 1A). Very interestingly this cardiac fibrosis reduction was not observed after transplantation of cADSC isolated from P2Y_2_ KO mice (Figures 1A,B). Effectively collagen stained area observed in PBS-injected ischemic mice (24.0 ± 1.3%; mean ± SEM), was comparable after P2Y_2_ KO ADSC transplantation (22.3 ± 1.0%; mean ± SEM). We observed comparable collagen blue staining for the fibrotic area of ischemic hearts injected with P2Y_2_ KO cADSC and PBS-injected ischemic hearts (Figure 1A, ×20 magnification). Collagen stainings are shown inside the fibrotic and remote areas of ischemic hearts injected with PBS, WT cADSCs or P2Y_2_ KO cADSC (Figure 1A, ×80 magnification).

The inhibitory effect of undifferentiated cADSC transplantation on post-ischemic cardiac fibrosis is thus dependent on their P2Y_2_ receptor expression. Reduced cardiac fibrosis was observed without any required pretreatment of cADSC. The release of endogenous extracellular nucleotides currently observed during ischemia could thus regulate the cardioprotective action of transplanted cADSC through activation of P2Y_2_ receptors expressed at their surface.

### Identification of UTP Target Genes in Mouse cADSC

**FIGURE 2:**
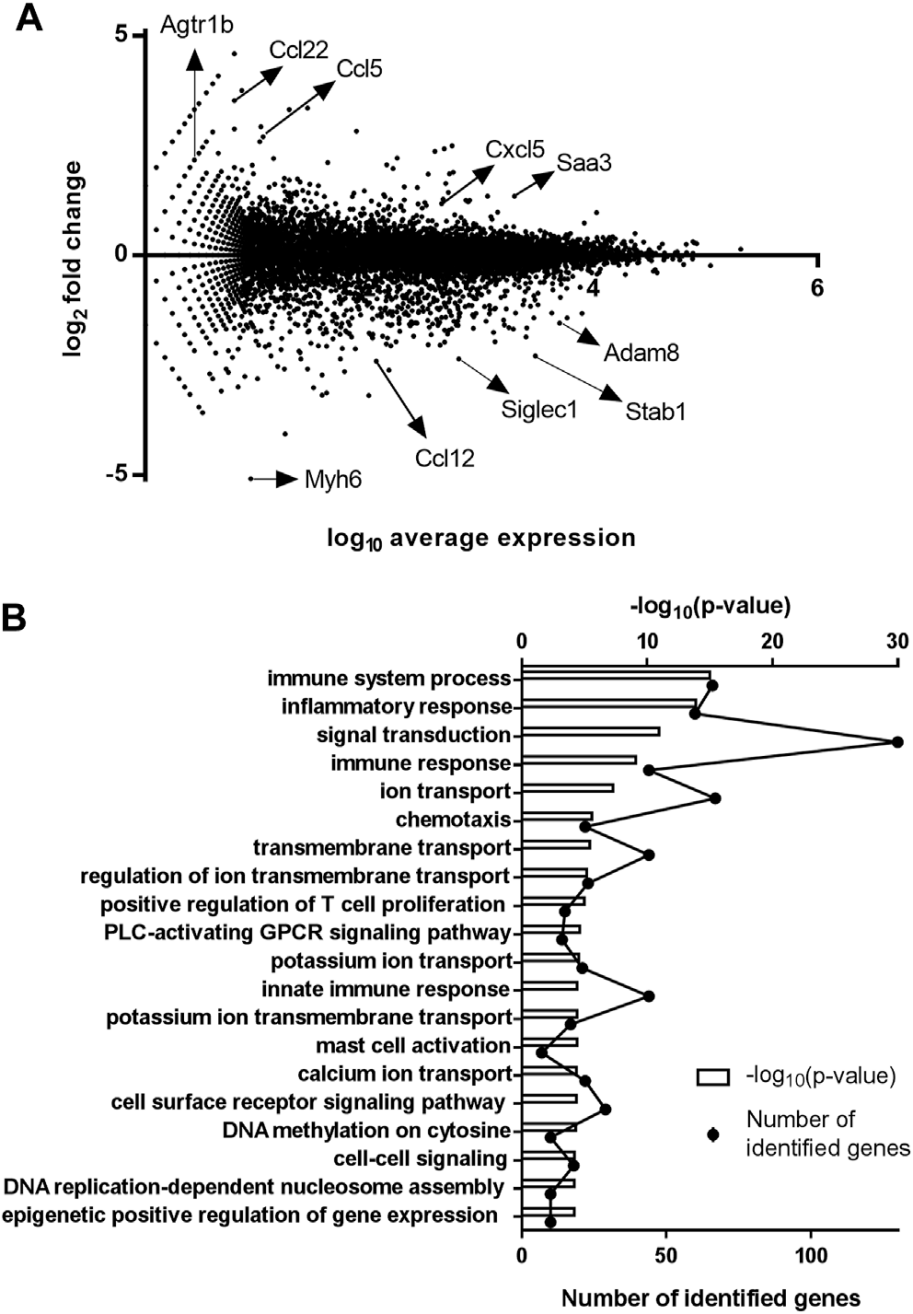
Identification of UTP target genes in mouse cADSC **(A)**. MA-plot of RNA-sequencing data comparing UTP-treated and untreated undifferentiated cADSC. RNA-sequencing experiments were performed on two RNA pools from two independent cultures of cADSC after 7 days in proliferation medium, and with or without daily stimulation with 100 µM UTP. Examples of upregulated genes (Agtr1b, Ccl5, Ccl22, Cxcl5, Saa3) and downregulated genes (Myh6, Ccl12, Siglec1, Stab1, Adam 8) are indicated by arrows. MA-plot shows the average expression on the X-axis and the log fold change on the *y*-axis of the two groups. MA plot; X = ln(√(x1*x2)), Y = log_2_(x1/x2). **(B)** Enriched biological processes related to UTP target genes in cADSC. Biological processes revealed as enriched for differentially expressed genes between UTP-treated and untreated cADSC after GO enrichment analysis performed with DAVID software (DAVID Bioinformatics Resources).

RNA-sequencing experiments were assessed on undifferentiated cADSC cells, unstimulated or treated daily with UTP (100 µM) during 7 days of culture in proliferation medium*.* RNA samples were extracted from two different cADSC cultures per condition and pooled for RNA-sequencing experiments. Among the 1,359 differentially regulated genes, 598 genes were upregulated by UTP (fold change > 2) and 761 were downregulated by UTP (fold change <0.5). MA-plot representation provides an overview of regulated-gene distribution within two-sample comparison (Figure 2A). We observed inter alia upregulation of Ccl5, Ccl22, Cxcl5, and downregulation of Ccl12 chemokine genes by UTP in ADSC (Figure 2A). A gene ontology (GO) enrichment analysis with DAVID software on the RNA-sequencing data obtained from UTP-treated ADSC versus untreated ADSC revealed that several biological processes linked to innate immune response, inflammation, as well as signal/receptor transduction and transmembrane/ion transport were enriched (Figure 2B). The modified Fisher Exact *p*-value or EASE score was reported by the software and indicated as logarithmic *p*-value.

### Detailed Analysis of Genes Regulated by UTP Through P2Y_2_ Receptor Activation in Mouse cADSC

We have previously demonstrated differential involvement of both mouse P2Y_2_ and P2Y_4_ receptors for UTP and ATP, in cADSC adipogenic differentiation thanks to P2Y_2_ and P2Y_4_ knockout mice (Lemaire et al., 2017; Negri et al., 2019). The absence of a clear detection of membrane-bound P2Y_2_ receptors remains a limitation. Our use of mouse monoclonal anti-P2Y_2_ antibody (H-5) from Santa Cruz Biotechnology (sc-518121) at 1:500 dilution was previously unsuccessful in mouse cADSC (data not shown). A comparable weak staining of cADSC was obtained with phycoerythrin-conjugated m-IgGκ secondary antibody alone (sc-516141; Santa Cruz Biotechnology) (data not shown).

We decided to compare UTP-regulated genes in WT and P2Y_2_ KO cADSC to identify more precisely genes and associated-biological processes specific of P2Y_2_ activation in these cells, and potentially linked to our cardiac fibrosis data using WT and P2Y_2_ KO ADSC (Figure 1). UTP is used as a stimulating agent instead of ATP to avoid regulation of genes related to ADP or adenosine receptor activation after ATP degradation. We have not detected expression of P2Y_6_ UDP receptor in cADSC (data not shown). A UTP-treatment during 7 days with UTP would thus activate only P2Y_2_ and P2Y_4_ receptors in cADSC.

**FIGURE 3:**
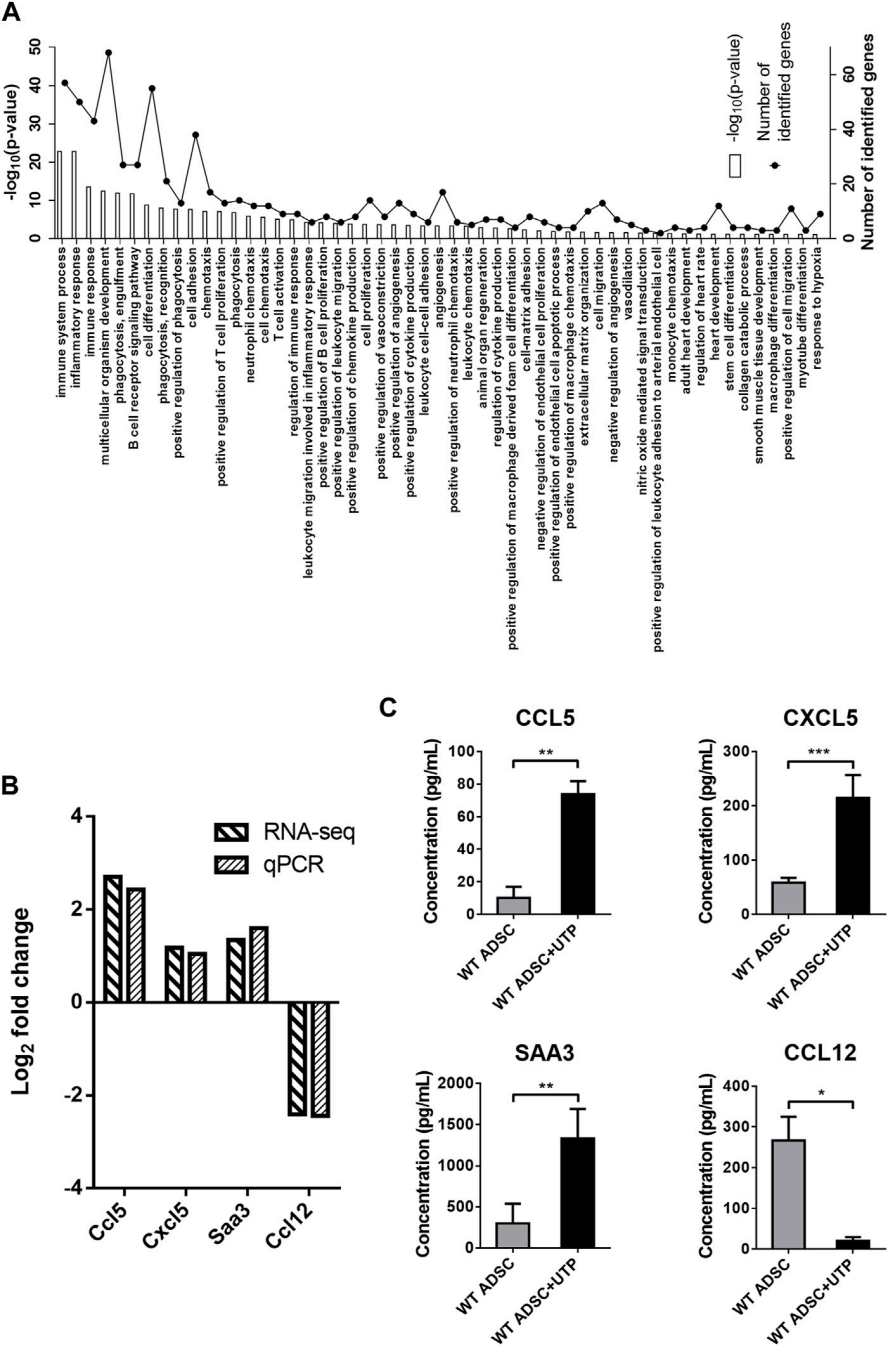
Detailed analysis of genes regulated by UTP through P2Y_2_ receptor activation in cADSC. **(A)** Enriched biological processes related to UTP target genes after comparison of RNA-sequencing data in WT and P2Y_2_ KO cADSC. RNA-sequencing experiments were performed on two RNA pools from two independent cultures of WT and P2Y_2_ KO cADSC after 7 days in proliferation medium and daily stimulated or not with 100 µM UTP. Comparison of RNA-sequencing data identified 977 genes regulated by UTP only in WT ADSC and not in P2Y_2_ KO cADSC. GO detailed analysis revealed various enriched biological processes related to the 977 UTP target genes. **(B)** Confirmation of specific UTP target genes by qPCR experiments. Ccl5, Cxcl5, and Saa3 mRNAs are upregulated, and Ccl12 mRNA is downregulated, in UTP-treated versus untreated cADSC in RNA-sequencing and qPCR experiments. Quantification of Ccl5, Cxcl5, Saa3, and Ccl12 mRNA level was performed by qPCR in at least six independent cADSC cultures and normalized to Rpl32 mRNA level. **(C)** UTP increases CCL5, CXCL5, and SAA3 release, and inhibits CCL12 release by cADSC. CCL5, CXCL5, SAA3, and CCL12 protein level was measured in the supernatants of cADSC cultures after 7 days in proliferation medium with or without daily stimulation with UTP 100 µM. ELISA data were obtained for six to nine independent cADSC cultures. Values represent mean ± SEM. *p < 0.05, **p < 0.01 and ***p < 0.001.

RNA-sequencing experiments were performed using P2Y_2_ KO cADSC, unstimulated or treated daily with UTP (100 µM). UTP gene expression profile was compared in both WT and P2Y_2_ KO cADSC*,* only genes with CPM >0.5 and a fold change >2 or <0.5 being considered. Among the 1,359 regulated genes identified in WT cADSC (Figure 2A), we observed that 977 genes were no more regulated in P2Y_2_ KO cADSC whereas 382 genes were still regulated by UTP in P2Y_2_ KO cADSC. A detailed GO enrichment analysis was made for these 977 UTP target genes only identified in WT ADSC and not in P2Y_2_ KO ADSC (Figure 3A). Besides the important number of genes involved in immune and inflammatory responses, GO analysis revealed enriched biological processes linked to multicellular organism development, cell differentiation and adhesion, angiogenesis and response to hypoxia (Figure 3A). Table 2 shows UTP target genes linked to processes moderately enriched but potentially related to our model of cardiac ischemia. Among the 977 UTP target genes, we identified genes linked to extracellular matrix organization and collagen catabolic process such as matrix metalloproteinases Mmp1b, Mmp9, and Mmp25 and collagen types Col10a1, Col17a1, and Col25a1, and genes related to macrophage chemotaxis such as Ccr7 chemokine receptor and colony-stimulating factor 1 receptor (Csf1r) (Table 2). We also identified UTP target genes involved in macrophage differentiation into pro-fibrotic foam cell such as Csf2, macrophage scavenger receptor Msr1 and apolipoprotein B (ApoB), as well as genes linked to response to hypoxia such as Agtr1b, purinergic receptor P2X_2_, adenosine A1 receptors and uncoupling protein Ucp3 (Table 2). Interestingly, we identified a significant number of UTP target genes linked to angiogenesis regulation such as Wnt7a, Ramp3, Mmp9, Nos3, and Adam8 (Table 2).

**TABLE 2:** Selection of genes regulated by UTP through P2Y_2_ receptor activation in cADSC. RNA-sequencing data comparison of UTP target genes in WT and P2Y_2_ KO cADSC, revealed 977 genes regulated by UTP only in WT ADSC and not in P2Y_2_ KO cADSC. Several processes and associated UTP target genes potentially linked to the studied model of cardiac ischemia, were identified after a detailed GO analysis.

Gene symbol	Gene name	Ratio
**UTP-regulated genes involved in extracellular matrix organization and collagen catabolic process**
Col17a1	Collagen type XVII. alpha 1 (Col17a1)	3.38
Mmp9	Matrix metallopeptidase 9 (Mmp9)	2.76
Col25a1	Collagen type XXV. alpha 1 (Col25a1)	2.00
Mmp1b	Matrix metallopeptidase 1b (interstitial collagenase) (Mmp1b)	2.00
Prtn3	proteinase 3 (Prtn3)	0.50
Adamts19	A disintegrin-like and metallopeptidase (reprolysin type) with thrombospondin type 1 motif. 19 (Adamts19)	0.48
Tgfbi	Transforming growth factor. beta induced (Tgfbi)	0.43
Mmp25	Matrix metallopeptidase 25 (Mmp25)	0.43
Tnf	Tumor necrosis factor (Tnf)	0.43
Col10a1	Collagen type X. alpha 1 (Col10a1)	0.19
Rxfp1	Relaxin/insulin-like family peptide receptor 1 (Rxfp1)	0.17
**UTP-regulated genes involved in macrophage chemotaxis and macrophage-derived foam cell differentiation**		
Csf2	Colony stimulating factor 2 (granulocyte-macrophage) (Csf2)	2.00
Apob	Apolipoprotein B (Apob)	2.00
Cebpe	CCAAT/enhancer binding protein (C/EBP). epsilon (Cebpe)	0.50
Spib	Spi-B transcription factor (Spi-1/PU.1 related) (Spib)	0.50
Pla2g5	Phospholipase A2. group V (Pla2g5)	0.50
Msr1	Macrophage scavenger receptor 1 (Msr1)	0.37
Csf1r	Colony stimulating factor 1 receptor (Csf1r)	0.37
C3ar1	Complement component 3a receptor 1 (C3ar1)	0.32
Ccr7	Chemokine (C-C motif) receptor 7 (Ccr7)	0.31
C5ar1	Complement component 5a receptor 1 (C5ar1)	0.26
**UTP-regulated genes involved in the response to hypoxia**		
Agtr1b	Angiotensin II receptor. type 1b (Agtr1b)	4.50
P2rx2	Purinergic receptor P2X. ligand-gated ion channel. 2 (P2rx2)	3.33
Chrna4	Cholinergic receptor. nicotinic. alpha polypeptide 4 (Chrna4)	2.78
Ascl2	Achaete-scute family bHLH transcription factor 2 (Ascl2)	2.00
Trh	Thyrotropin releasing hormone (Trh)	2.00
Ucp3	Uncoupling protein 3 (mitochondrial. proton carrier) (Ucp3)	2.00
Casp1	Caspase 1 (Casp1)	0.47
Adora1	Adenosine A1 receptor (Adora1)	0.44
Myb	Myeloblastosis oncogene (Myb)	0.42
**UTP-regulated genes involved in the regulation of angiogenesis**		
Wnt7a	Wingless-type MMTV integration site family. member 7A (Wnt7a)	4.14
Ramp3	Receptor (calcitonin) activity modifying protein 3 (Ramp3)	2.92
Mmp9	Matrix metallopeptidase 9 (Mmp9)	2.76
Nos3	Nitric oxide synthase 3. endothelial cell (Nos3)	2.07
Cnmd	Chondromodulin (Cnmd)	2.00
Il17f	Interleukin 17F (Il17f)	2.00
Nrxn3	Neurexin III (Nrxn3)	0.50
Shh	Sonic hedgehog (Shh)	0.50
Arhgap22	Rho GTPase activating protein 22 (Arhgap22)	0.49
Pik3r6	Phosphoinositide-3-kinase regulatory subunit 5 (Pik3r6)	0.47
Lepr	Leptin receptor (Lepr)	0.45
Hhex	Hematopoietically expressed homeobox (Hhex)	0.45
Cxcr3	Chemokine (C-X-C motif) receptor 3 (Cxcr3)	0.44
Plxdc1	Plexin domain containing 1 (Plxdc1)	0.44
Alox5	Arachidonate 5-lipoxygenase (Alox5)	0.43
Tgfbi	Transforming growth factor. beta induced (Tgfbi)	0.43
Tal1	T cell acute lymphocytic leukemia 1 (Tal1)	0.43
Cysltr1	Cysteinyl leukotriene receptor 1 (Cysltr1)	0.42
Cx3cr1	Chemokine (C-X3-C motif) receptor 1 (Cx3cr1)	0.41
Cysltr2	Cysteinyl leukotriene receptor 2 (Cysltr2)	0.40
Vav3	Vav 3 oncogene (Vav3)	0.39
Cd40	CD40 antigen (Cd40)	0.35
Adam8	A disintegrin and metallopeptidase domain 8 (Adam8)	0.34
Mir27b	microRNA 27b (Mir27b)	0.33
C3ar1	Complement component 3a receptor 1 (C3ar1)	0.32
Pik3cg	Phosphatidylinositol-4.5-bisphosphate 3-kinase catalytic subunit gamma (Pik3cg)	0.32
C5ar1	Complement component 5a receptor 1 (C5ar1)	0.26
Angptl3	Angiopoietin-like 3 (Angptl3)	0.25
C6	Complement component 6 (C6)	0.25
Thbs4	Thrombospondin 4 (Thbs4)	0.25
Ccl12	Chemokine (C-C motif) ligand 12 (Ccl12)	0.19

### UTP Induces CCL5, CXCL5 and SAA3 Secretion and Inhibits CCL12 Secretion by cADSC

Among all the UTP-regulated genes, we studied further the up-regulation of three pro-angiogenic genes, Ccl5, Cxcl5, and Saa3, and the down-regulation of the pro-fibrotic chemokine Ccl12. We confirmed up-regulation of Ccl5, Cxcl5, and Saa3, and downregulation of Ccl12 mRNAs in response to UTP in cADSC by qPCR experiments (Figure 3B). CCL5, CXCL5, CCL12, and SAA3 protein levels were quantified by ELISA in the supernatants of UTP-treated or untreated undifferentiated cADSC. We observed that UTP treatment increased the release of CCL5, CXCL5, and SAA3, and decreased the release of CCL12 by cADSC (Figure 3C).

SAA3 is a key inflammatory adipocyte-derived factor which is the murine ortholog of human serum amyloid A promoting angiogenesis in many diseases. (Lv et al., 2016; Tannock et al., 2018) Interestingly, CCL5 chemokine was previously reported as a major regulator of ADSC migration and post-ischemic reparative capacities. (Kauts et al., 2013; Kimura et al., 2014) UTP-mediated release of CCL5, CXCL5, and SAA3 by cADSC, as well as the consistent number of UTP-regulated genes involved in angiogenesis regulation (Table 2), led us to investigate the potential action of UTP-treated cADSC transplantation on cardiac revascularization.

### Effect of Transplanted UTP-Treated cADSC on Post-ischemic Cardiac Revascularization

**FIGURE 4:**
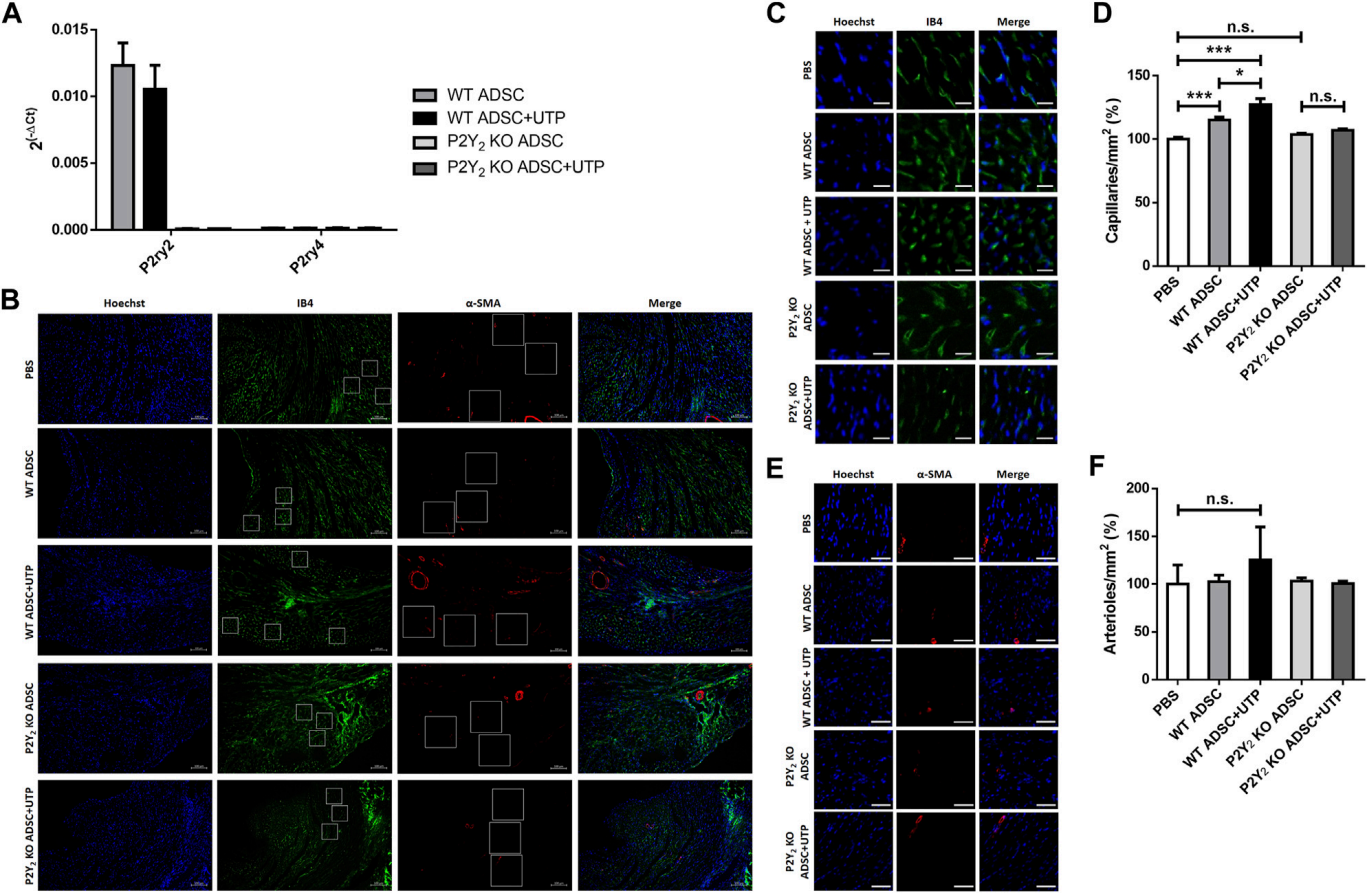
Effect of transplanted UTP-treated cADSC on revascularization of ischemic hearts. **(A)** Expression of P2Y_2_ and P2Y_4_ receptor mRNA in cADSC used for transplantation. mRNA expression level of P2Y_2_ and P2Y_4_ receptors was evaluated in cADSCs after 7 days of culture in the presence or the absence of 100 µM UTP by quantitative PCR experiments. The data have been normalized to RPL32 mRNA expression. **(B)** Immunofluorescence images of Isolectin B4 (IB4) (green), α-Smooth Muscle Actin (α-SMA) (red) and Hoechst (blue) in the peri-infarct border zone of ischemic hearts, 14 days after injection of PBS, WT or P2Y_2_ KO cADSC untreated or treated with UTP (100 µM). Representative counting surface/field of capillary density quantification, based on IB4-staining (green), and representative counting surface/field of arteriole density quantification, based on α-SMA-staining (red) are indicated by squares. Scale bar represents 100 µm (×10 magnification). **(C)** Representative counting surface (0.1 mm^2^)/field of capillary density quantification in IB4 (green) and Hoechst (blue) stained hearts sections. Scale bar represents 20 µm. **(D)** Capillary density quantification, based on IB4-staining, 14 days after injection of PBS, untreated or UTP-treated WT or P2Y_2_ KO cADSC in ischemic hearts. Capillary density was calculated in 30 counting surfaces per ischemic heart. **(E)** Representative counting surface (0.4 mm^2^)/field of arteriole density in α-SMA (red) and Hoechst (blue) stained heart sections. Scale bar represents 50 µm. **(F)** Arteriole density quantification, based on α-SMA (red)/IB4 (green) staining, 14 days after injection of PBS, untreated or UTP-treated WT or P2Y_2_ KO cADSC in ischemic hearts. Arteriole density was calculated in 30 counting surfaces per ischemic heart. Values are presented as mean ± SEM (*n* = 5–8 mice per each group). *p < 0.05, **p < 0.01, ***p < 0.001, and n.s., not significant.

WT and P2Y_2_ KO cADSC were cultured without or with daily stimulation of UTP (100 µM) for 7 days in proliferation medium. Flow cytometry experiments revealed a comparable amount, between 92% and 95%, of CD90^+^ CD105^+^ CD44^+^ CD26^−^ cells in all the cell cultures (data not shown). We have performed qPCR experiments to quantify P2Y_2_ and P2Y_4_ mRNA in wild type cADSC, after stimulation or not with 100 µM during 7 days (Figure 4A). We observed a high level of expression of P2Y_2_ mRNAs compared to weakly detected P2Y_4_ receptor mRNAs, in cADSC after 7 days of culture in the proliferation medium (Figure 4A). We also observed that sustained stimulation with 100 µM UTP during 7 days did not induce down-regulation of P2Y_2_ receptor mRNAs (Figure 4A).

Intramyocardial injections of PBS (control), untreated WT or P2Y_2_ KO cADSC, and UTP-treated WT or P2Y_2_ KO cADSC were performed in ischemic mice, 3 days after LAD ligation. Immunofluorescent stainings using anti-isolectin B4 and anti-α-SMA antibodies were performed on heart sections, 14 days after cASC injection, to quantify capillary and arteriole density respectively, in infarct border zone at different levels of the ischemic heart (Figure 4B). Analysis of peri-infarct microvasculature 14 days after cADSC injection revealed a higher capillary density in heart transplanted with untreated cADSC compared to PBS-injected mice (increase of 15.2 ± 2.8%; mean ± SEM; ***: *p* < 0.001) (Figures 4C,D). Capillary density was even more enhanced in heart transplanted with UTP-treated cADSC compared to PBS-injected mice (increase of 27.2 ± 4.2%; mean ± SEM; ***: *p* < 0.001) (Figure 4D). Interestingly, capillary density was similar in P2Y_2_ KO cADSC-transplanted mice and in PBS-injected mice (Figure 4D). Moreover, the effect of UTP treatment on the capacity of cADSC to increase capillary density, was not observed using UTP-treated P2Y_2_ KO cADSC (Figure 4D). No significant effect of UTP-treated or untreated ADSC transplantation was observed on arteriole density, defined by the number of α-SMA positive vessels with a diameter <20 µm (Figures 4E,F).

## Discussion

Therapeutic strategies contributing to myocardial repair by regulating cardiac fibrosis and revascularization still need to be improved. Early reperfusion by restoration of blood flow is the way to heal the ischemic myocardium by limiting infarct size and improving clinical outcomes (Zhao et al., 2017). Multiple challenges have to be solved to establish a routine clinical use of ADSC-based cell therapy. The regulatory function of cardiac adipose tissue in cardioprotection represents a major interest by its proximity to the myocardium. Despite its limited size, cardiac adipose tissue was described as an ideal source of therapeutically effective ADSC for cardiac regeneration (Nagata et al., 2016). cADSC have a higher proliferation activity *in vitro* than ADSC isolated from other sources, such as visceral, subcutaneous and subscapular adipose tissues (Nagata et al., 2016). Systemic transfusion of cADSC in ischemic mice leads to the highest cardiac functional recovery after myocardial infarction, compared to other types of ADSC (Nagata et al., 2016).

We have previously demonstrated that UTP regulates the endothelial differentiation and the angiogenic effects of cADSC (Vanorlé et al., 2021). This previous work was exclusively based on the use of UTP-treated cADSC differentiated into endothelial cells, to regulate post-ischemic revascularization (Vanorlé et al., 2021). The present study investigates the potential involvement of P2Y_2_ on the beneficial effects of undifferentiated cADSC injection on post-ischemic cardiac fibrosis and revascularization. Undifferentiated cADSC were used here to study the consequence of their injection into mouse ischemic heart without any orientation into a specific cell lineage. Their effect on both post-ischemic cardiac fibrosis and revascularization was observed without a required pretreatment of cADSC with extracellular nucleotides. Interestingly, the inhibitory effect of undifferentiated cADSC transplantation on cardiac fibrosis was no more observed using P2Y_2_ KO cADSC. The release of endogenous extracellular nucleotides on the ischemic site could thus contribute to regulate the cardioprotective action of transplanted cADSC through activation of P2Y_2_ receptors expressed at their surface.

RNA-sequencing experiments revealed a gene expression profile very different in UTP-treated undifferentiated cADSC, than the one we had previously reported in UTP-treated cADSC differentiated into endothelial cells (Vanorlé et al., 2021). We have previously demonstrated differential involvement of both P2Y_2_ and P2Y_4_ receptors in cADSC adipogenic differentiation (Lemaire et al., 2017; Negri et al., 2019). We compared here UTP-regulated genes in WT and P2Y_2_ KO cADSCs to discriminate genes and associated-biological processes specific of P2Y_2_ activation in these cells. Detailed gene profiling analysis revealed UTP-regulated genes linked to extracellular matrix organization and collagen catabolism such as matrix metalloproteinases and many collagen types, and UTP-regulated genes related to macrophage chemotaxis and foam cell differentiation. We also demonstrated UTP-mediated inhibition of CCL12 release by ADSC. CCL12 is a pro-fibrotic chemokine that generates an unfavorable cardiac healing environment (DeLeon-Pennell et al., 2017). The inhibition of CCL12 release by extracellular nucleotides present on the ischemic site could contribute to ADSC inhibitory action on cardiac fibrosis, and ameliorate cardiac repair. Ccl22 was also identified as a UTP target gene in ADSC in our RNA-sequencing experiments. CCL22 is involved in CCR4-mediated cardiac cell migration (Safa et al., 2016) and CCL22/CCR4 polymorphisms have been associated to patients diagnosed with myocardial infarction (Noori et al., 2018).

Interestingly, a significant number of UTP-regulated genes in ADSC are linked to angiogenesis regulation. More precisely, we showed that UTP increased pro-angiogenic Ccl5, Cxcl5, and Saa3 mRNAs by qPCR experiments, and the release of CCL5, CXCL5, and SAA3 in the supernatants of undifferentiated ADSC. The release of CCL5/RANTES by cADSC in response to UTP was particularly interesting. CCL5 is an angiogenic chemokine, and CCL5/CCR5 axis is notably involved in VEGF-mediated tumor angiogenesis (Wang et al., 2015). CCL5 was also reported to stimulate multipotency, migration and post-ischemic reparative capacities of ADSC (Kimura et al., 2014). The fact that transplanted ADSC have higher repair capacities of the ischemic tissue than bone marrow or dental tissue mesenchymal cells was associated to their higher secretion of CCL5 chemokine (Kimura et al., 2014). ADSC were the most effective stem cells to increase microvessel formation and ADSC lacking CCL5 expression were no more able to restore blood flow in the ischemic limb model (Kimura et al., 2014).

A high expression of P2Y_2_ receptor mRNAs was detected in unstimulated and UTP-treated cADSC used for transplantation, compared to low P2Y_4_ receptor mRNA expression. P2Y_4_ receptor is involved in cADSC adipogenic differentiation (Lemaire et al., 2017) but its expression in these cells was effectively very low after 7 days of culture in the proliferation medium. We used UTP instead of ATP to prestimulate cADSC to avoid ADP or adenosine receptor activation due to ATP degradation. We confirmed that prolonged UTP stimulation during 7 days did not induce P2Y_2_ receptor desensitization before transplantation. Quantification of capillary and arteriole density in the ischemic border zone revealed that, 14 days after intramyocardial transplantation of WT cADSC, a higher capillary density was found around the necrotic zone. This effect was enhanced by a pretreatment of cADSC with UTP before transplantation. Interestingly this positive effect on post-ischemic revascularization was lost using P2Y_2_ KO cADSC. These data showed that the orientation of cADSC differentiation into endothelial cells was not necessary to have an effect of their transplantation on post-ischemic revascularization and highlight the importance of P2Y_2_ expression in their capacity to increase capillary density.

Extracellular nucleotide-mediated CCL5 secretion by cADSC in the ischemic heart could contribute to their beneficial action on cardiac revascularization and fibrosis after transplantation. Neutralizing anti-CCL5 antibodies have provided a therapeutic benefit in a mouse model of chronic cardiac ischemia (Montecucco et al., 2012). Nevertheless targeting CCR5 was not always effective in cardiovascular treatments because CCL5/CCR5 facilitates progenitor cell recruitment and promotes vascular endothelial cell repair (Zhang et al., 2020). CCR5 signaling is reported to suppress inflammation and reduce adverse remodeling of the infarcted heart (Dobaczewski et al., 2010).

A P2Y_2_-mediated cardioprotective effect of UTP injection was described in ischemic mice. Cohen et al. (2011) described that UTP injection reduces infarct size and improves cardiac function after myocardial infarct in mice through P2Y_2_ activation of cardiomyocytes (Cohen et al., 2011). Therapeutic use of UTP intramyocardial injection is problematic, knowing its rapid degradation and the ubiquitous expression of P2Y_2_ receptor, accompanied by multiple side effects on cardiac and immune cells. The activation of P2Y_2_ receptors and its target proteins in cADSC before their transplantation would be an appropriate way to amplify their beneficial effects and ameliorate post-ischemic cardiac response. This therapeutic approach would avoid the problems inherent to intramyocardial injection of extracellular nucleotides which are due to their rapid metabolism and ubiquitous receptor expression.

Injected stem cells are known to be rapidly eliminated after their injection in the ischemic heart, limiting strongly their possible integration into the fibrotic area or in newly formed capillaries (Schenke-Layland et al., 2009; Zhao et al., 2017; Vanorlé et al., 2021). The restricted engraftment of intramyocardial transplanted stem cells points out the importance of their paracrine effects (Patel and Zhang, 2014). A lot of inflammatory mediators including extracellular nucleotides, chemokines and angiogenic factors, are released in the infarct zone and can modulate the action of injected cADSC. Extracellular nucleotides could contribute to the regulation of cADSC paracrine signals, such as the secretion of pro-angiogenic chemokines and other target proteins identified in our RNA-sequencing experiments. The supernatant of cADSC is removed for cell counting before injection in the ischemic heart, but their pretreatment with UTP changes their gene expression profile and amplifies their effects on cardiac revascularization. Strategies using stem cell preconditioning are known to improve their paracrine ability to release cardioprotective factors (Baraniak and McDevitt, 2010). The identification of UTP target genes in cADSC involved in cardiac angiogenesis and fibrosis, is very promising and needs further investigation to define all the involved mechanisms.

Altogether, our data indicate that the activation of nucleotide receptors on the surface of cADSC exerts a prevalent action on their cardioprotective abilities. The present study defines P2Y_2_ receptor as a key regulator of the therapeutic use of undifferentiated cADSC against cardiac ischemia, and provides a novel insight for the optimization of cardiac repair cell therapies.

## Data Availability

The datasets presented in this study can be found in online repositories. The names of the repository/repositories and accession number(s) can be found below: https://www.ncbi.nlm.nih.gov/geo/query/acc.cgi?acc=GSE201055.
